# Influence of machine learning membership functions and degree of membership function on each input parameter for simulation of reactors

**DOI:** 10.1038/s41598-021-81514-y

**Published:** 2021-01-21

**Authors:** Rasool Pelalak, Ali Taghvaie Nakhjiri, Azam Marjani, Mashallah Rezakazemi, Saeed Shirazian

**Affiliations:** 1 Institute of Research and Development, Duy Tan University, Da Nang, 550000 Viet Nam; 2 Faculty of Environmental and Chemical Engineering, Duy Tan University, Da Nang, 550000 Viet Nam; 3 Department of Petroleum and Chemical Engineering, Science and Research Branch, Islamic Azad University, Tehran, Iran; 4 Department for Management of Science and Technology Development, Ton Duc Thang University, Ho Chi Minh City, Viet Nam; 5 Faculty of Applied Sciences, Ton Duc Thang University, Ho Chi Minh City, Viet Nam; 6 Faculty of Chemical and Materials Engineering, Shahrood University of Technology, Shahrood, Iran; 7 Laboratory of Computational Modeling of Drugs, South Ural State University, 76 Lenin Prospekt, Chelyabinsk, Russia 454080

**Keywords:** Chemical engineering, Mechanical engineering, Computational science

## Abstract

To understand impact of input and output parameters during optimization and degree of complexity, in the current study we numerically designed a bubble column reactor with a single sparger in the middle of the reactor. After that, some input and output parameters were selected in the post-processing of the numerical method, and then the machine learning observation started to investigate the level of complexity and impact of each input on output parameters. The adaptive neuro-fuzzy inference system (ANFIS) method was exploited as a machine learning approach to analyze the gas–liquid flow in the reactor. The ANFIS method was used as a machine learning approach to simulate the flow of a 3D (three-dimensional) bubble column reactor. This model was also used to analyze the influence of input and output parameters together. More specifically, by analyzing the degree of membership functions as a function of each input, the level of complexity of gas fraction was investigated as a function of computing nodes (X, Y, and Z directions). The results showed that a higher number of membership functions results in a better understanding of the process and higher model accuracy and prediction capability. X and Y computing nodes have a similar impact on the gas fraction, while Z computing points (height of reactor) have a uniform distribution of membership function across the column. Four membership functions (MFs) in each input parameter are insufficient to predict the gas fraction in the 3D bubble column reactor. However, by adding two membership functions, all features of gas fraction in the 3D reactor can be captured by the machine learning algorithm. Indeed, the degree of MFs was considered as a function of each input parameter and the effective parameter was found based on the impact of MFs on the output.

## Introduction

Bubble column type of chemical reactors are mainly employed and applied in different chemical, wastewater, oil, and gas industries to produce the interaction between various phases, such as gas, liquid, and solid phases for the purpose of either reactions or separations^1–4^. This multiphase interaction can be a great domain for chemical and physical interactions and heat and mass transfer between different reactors, resulting in separation in wastewater treatment technology or generation of one product in the oil and gas industries^2,5^. Because of this reactor's simple shape, less mixing tools, low rate of maintenance, and high rate of heat and mass transfer in the domain, they are preferred over other reactors in industrial and research activities. They have a simple structure for capturing all physical interactions, such as bubble break-up or coalescence of bubbles by simple Process Analytical Technology (PAT) such as high-speed cameras^1,2,5,6^.

These bubbly reactors are also equipped with a sparger at the bottom of domain to inject different types of gas in the reactor. The sparger specifications can mainly change the regime of flow and rate of interaction between phases^2,7,8^. All specifications, such as sparger size, arrangement, pattern, and gas velocity at the sparger entrance, can impact the flow regime in the bubble column reactor^8–11^. For instance, a low rate of gas entrance velocity can produce a homogeneous flow regime with almost tiny air/gas bubbles. By increasing this velocity; gas bubbles intend to merge together, called coalescence of bubble and the changes from homogeneous flow to heterogeneous flow regime. At some point in the high rate of gas sparger into the domain, bubbles are fully connected together, and the regime of flow near the sparger can be turbulence, and bubbles are fully merged together and challenging, or jet flow occurs in the reactor^12^. At this stage, bubble detachment rarely is happened, and few bubbles coalesce together, and then they detach from the tip of sparger.

Many numerical and experimental studies focus on the influence of sparger specification on the bubble column hydrodynamics^8–10^. They mainly concentrated on superficial gas velocity, sparger arrangement, and types of sparger. Computational fluid dynamics (CFD) is one of the main tools to calculate the influence of sparger specification on the gas and liquid flow pattern. This numerical toolbox by solving Navier–Stokes equations can provide the local calculation of several flow characteristic parameters in different parts or local positions of the reactor. The local calculation, such as velocity or fraction of gas/liquid components at each computing point, enables the researchers to optimize the flow in the reactor more accurately^3^. However, getting local flow in the experimental study can be very time-consuming and expensive.

The CFD calculation can always provide a deep insight into the bubble column reactor^5,7,13–17^, but as many parameters are solved through CFD analysis, understanding all parameters requires intensive time with knowledge of statistics and engineering. Additionally, the connection between each input and output parameter can be another challenge for the researchers. Finding the main effective parameters in the process is always important to analyze and understand the process, which impacts the design and optimization of these types of reactors^18^. Machine learning-based approaches have recently been extensively developed to model different processes, such as bubbly flow in bubble column reactors or heat and mass transfer problems. They suggested different algorithms to train CFD data set, and then predict process, such as adaptive neuro-fuzzy inference system (ANFIS) and ant colony optimization (ACO) plus fuzzy system. They showed that their models are accurate in predicting the CFD data set, and it can find the pattern of flow in the domain^19,20^. However, there is a lack of analysis for the impact of each parameter on the output of the CFD calculation. Additionally, there is a need to understand the behavior of function specifications in each input.

In this study, the location of the sparger from the bottom to the middle of the reactor was changed by using the CFD method (Eulerian framework), and then with the ability of machine learning methods, the flow was mimicked in the reactor. The computed amount of gas fraction at CFD elements participated in the ANFIS method for training and evaluation (testing). Then, the generated machine learning method through the CFD data set was used for more analysis and understanding of the process. The function analysis was applied to determine the complexity of each input parameter on the gas–liquid flow pattern. Additionally, the ability of the prediction process was elaborated for a different number of membership functions for each input. The machine learning method was applied to understand the procedure in the study better; also, the process was facilitated by having a mathematical correlation between the parameters. The current research study is novel as far as in previous studies the main focus had been on the effect of inputs on the output. However, the current study sought to investigate studies which input had the highest effect on the output. The effective parameter can be found based on the impact of MFs on the output, and therefore, the degree of MFs was considered as a function of each input parameter. This would provide a high-performance simulation methodology compared to conventional models for chemical reactors.

## Methods

### CFD

The 3D bubble column reactor was simulated by CFD methods. For modeling of sparger, the source point was considered in the domain with velocity in z-direction equal 2 m/s. This source point sparger illustrates a single sparger in the domain. The sparger location was considered in the middle of the reactor to observe the impact of the sparger location when it was located in the middle of the reactor. The bubble for this simulation was 3 mm throughout the domain, and this size of the bubble represented a homogeneous flow regime with uniform spherical bubbles in the reactor. Air bubble sparger through single-sized sparger produced a dispersed phase with 20 degrees, while the continuous phase is water.

For solving the fluid flow in the bubble column reactor, the Eulerian method was applied in the study. In this method, the gas sparger was modeled by the source point. Furthermore, the fluid in the bubble column reactor was considered as a continuous fluid. As shown in Eq. 1, the interaction between the gas and liquid phases was considered by using the K term, which shows the effect of gas on the liquid phase changing in time:

In this equation, ***u***_*k*_ and *ϵ*_*k*_ are the volume fraction and velocity of the fluid for each of the phases, including gas and liquid phases^8,11,21^:

1 $$\frac{\partial }{{\partial {\text{t}}}}\left( {{\uprho }_{{\text{k}}} \epsilon_{{\text{k}}} } \right) + \nabla \cdot \left( {{\uprho }_{{\text{k}}} \epsilon_{{\text{k}}} {\mathbf{u}}_{{\text{k}}} } \right) = { }0$$

where ${\mathbf{u}}_{{\text{k}}}$ represents the average calculation of velocity for phase k (both dispersed and continuous phases). The fraction of gas/liquid in the domain and density of both phases are defined as $\epsilon_{{\text{k}}}$ and ${\uprho }_{{\text{k}}}$, respectively.

Equation 2 is the momentum equation in which different terms are seen, including stress, pressure, gravity and interfacial force model terms. For the interfacial force term, the drag force equations are considered^7,8,22^:

2 $$\frac{\partial }{{\partial {\text{t}}}}\left( {{\uprho }_{{\text{k}}} \epsilon_{{\text{k}}} {\mathbf{u}}_{{\text{k}}} } \right) + \nabla \cdot \left( {{\uprho }_{{\text{k}}} \epsilon_{{\text{k}}} {\mathbf{u}}_{{\text{k}}} {\mathbf{u}}_{{\text{k}}} } \right) = - \nabla \cdot \left( {\epsilon_{{\text{k}}} {\uptau }_{{\text{k}}} } \right) - \epsilon_{{\text{k}}} \nabla {\text{P}} + \epsilon_{{\text{k}}} {\uprho }_{{\text{k}}} {\text{g}} + {\mathbf{M}}_{{{\text{I}},{\text{k}}}} { }$$

In this equation, $P, \tau g,$ and ${\mathbf{M}}_{{{\text{I}},{\text{k}}}}$ describe the pressure, stress, gravity and interfacial force models.

Also, the stress term could be considered for the k phase in Eq. 3, which is as follows^12,23^:

3 $${\uptau }_{{\text{k}}} = - {\upmu }_{{{\text{eff}},{\text{k}}}} \left( {\nabla {\mathbf{u}}_{{\text{k}}} + \left( {\nabla {\mathbf{u}}_{{\text{k}}} } \right)^{{\text{T}}} - \frac{2}{3}{\mathbf{I}}\left( {\nabla \cdot {\mathbf{u}}_{{\text{k}}} } \right)} \right){ }$$

Here, ${\upmu }_{{{\text{eff}},{\text{k}}}}$ is effective phase viscosity, which could be divided into three different terms represented in Eq. 4^7,8,12,19,23^:

4 $${\upmu }_{{{\text{eff}},{\text{L}}}} = {\upmu }_{{\text{L}}} + {\upmu }_{{{\text{T}},{\text{L}}}} + {\upmu }_{{{\text{BI}},{\text{L}}}} { }$$

The molecular viscosity, the liquid turbulent viscosity, and the turbulent viscosity are explained in the equation by *μ*_*L*_, *μ*_*T,L,*_and *μ*_*BI,L*_. The effective gas viscosity can also be considered in Eq. 5, which is as follows, and it could be a function of gas and liquid density^12,22–24^:

5 $${\upmu }_{{{\text{eff}},{\text{G}}}} = \frac{{{\uprho }_{{\text{G}}} }}{{{\uprho }_{{\text{L}}} }}{\upmu }_{{{\text{eff}},{\text{L}}}} { }$$

The total interfacial force is then estimated as^8^:

6 $${\mathbf{M}}_{{{\text{I}},{\text{L}}}} = - {\mathbf{M}}_{{{\text{I}},{\text{G}}}} = {\mathbf{M}}_{{{\text{D}},{\text{L}}}} + {\mathbf{M}}_{{{\text{TD}},{\text{L}}}} { }$$

${\mathbf{M}}_{{{\text{D}},{\text{L}}}}$ and ${\mathbf{M}}_{{{\text{TD}},{\text{L}}}}$ represent drag and turbulent dispersion force models.

The interphase momentum transfer between two phases, including gas and liquid, is as^8^:

7 $${\mathbf{M}}_{{{\text{D}},{\text{L}}}} = - \frac{3}{4} \in_{{\text{G}}} {\uprho }_{{\text{L}}} \frac{{{\text{C}}_{{\text{D}}} }}{{{\text{d}}_{{\text{B}}} }}\left| {{\mathbf{u}}_{{\text{G}}} - {\mathbf{u}}_{{\text{L}}} } \right|\left( {{\mathbf{u}}_{{\text{G}}} - {\mathbf{u}}_{{\text{L}}} } \right)$$

In this equation ${\text{C}}_{{\text{D}}}$ and ${\text{d}}_{{\text{B}}}$ shows drag coefficient and bubble size in the numerical calculation^20^.

According to the equations that exist in the momentum equation, there is an interfacial force model in which the drag model is only considered, and turbulent dispersion force is considered. Drag force equation was modeled by using the Schiller-Neumann formula^24^.

In the Schiller-Naumann drag model, the bubbles were considered spherical and fixed. Also, it is hypothesized that the interaction among the bubbles is very low. Furthermore, as the literature shows, this equation is highly acceptable and suitable for spherical and uniform bubbles^8^. The turbulent dispersion force was also considered in the equations for solving the movements of the bubbles in the fluid. Turbulent diffusion of the bubbles is as follows^8,23^:

8 $${\mathbf{M}}_{{{\text{TD}},{\text{L}}}} = - {\mathbf{M}}_{{{\text{TD}},{\text{G}}}} = - {\text{C}}_{{{\text{TD}}}} {\uprho }_{{\text{L}}} {\text{k}}\nabla \in_{{\text{L}}}$$

where *k* and *C*_*TD*_ show the kinetic energy and model parameter. On the other hand, for solving the turbulent characteristics in the bubble column reactor, the k-epsilon model is used, which is a reliable model in industries. The model can present an acceptable solving based on the average estimation of the velocity. Moreover, comparing this model to other turbulence models, the model includes low-costs.

As shown in Eq. 9, turbulent eddy viscosity is defined for the multiphase flow below:

9 $${\upmu }_{{{\text{T}},{\text{L}}}} = {\uprho }_{{\text{L}}} {\text{C}}_{{\upmu }} \frac{{{\text{k}}^{2} }}{{\upvarepsilon }}{ }$$

Equations 10 and 11, energy dissipation rate (*ε*), as well as the turbulent kinetic energy (k), are shown below^8,11,19,22,23^:

10 $$\frac{\partial }{{\partial {\text{t}}}}\left( {{\uprho }_{{\text{L}}} \in_{{\text{L}}} {\text{k}}} \right) + \nabla \left( {{\uprho }_{{\text{L}}} \in_{{\text{L}}} {\text{u}}_{{\text{L}}} {\text{k}}} \right) = - \nabla \left( { \in_{{\text{L}}} \frac{{{\upmu }_{{{\text{eff}},{\text{L}}}} }}{{{\upsigma }_{{\text{k}}} }}\nabla {\text{k}}} \right) + \in_{{\text{L}}} \left( {{\text{G}} - {\uprho }_{{\text{L}}} {\upvarepsilon }} \right){ }$$

11 $$\frac{\partial }{{\partial {\text{t}}}}\left( {{\uprho }_{{\text{L}}} \in_{{\text{L}}} {\upvarepsilon }} \right) + \nabla \left( {{\uprho }_{{\text{L}}} \in_{{\text{L}}} {\text{u}}_{{\text{L}}} {\upvarepsilon }} \right) = - \nabla \left( { \in_{{\text{L}}} \frac{{{\upmu }_{{{\text{L}},{\text{eff}}}} }}{{{\upsigma }_{{\upvarepsilon }} }}\nabla {\upvarepsilon }} \right) + \in_{{\text{L}}} \frac{{\upvarepsilon }}{{\text{k}}}\left( {{\text{C}}_{{{\upvarepsilon }1}} {\text{G}} - {\text{C}}_{{{\upvarepsilon }2}} {\uprho }_{{\text{L}}} {\upvarepsilon }} \right){ }$$

In these equations, $C\mu = 0.09$, $\sigma_{k} = 1$, $\sigma_{\varepsilon } = 1$, $C_{\varepsilon 1} = 1.44$, and $C_{\varepsilon 2} = 1.92$ are fixed model parameters within the frame of $k$-turbulence model.

### Adaptive-network-based fuzzy inference system (ANFIS)

The ANFIS (Adaptive-Network-based Fuzzy Inference System) was used for solving the equations in the AI. Three inputs exist, which are x, y, and z computing nodes. Furthermore, the output is the gas fraction of each of the elements in the bubble column reactor in this computational study^27^. The ANFIS model has the capability for solving, which comes from the learning process of the neural network and the decision stage of the fuzzy logic system.

Due to the various functions and capability of the model for introducing new functions for each of the inputs, the method enables the researchers for suitable learning for different data sets^11,25^.

In this method, the incoming signal from the first layer is multiplied based on AND rule. After that, the signals enter the second layer^11^.

As shown in Eq. 12, all the inputs are considered exchanging among the signals between the layers.

12 $$w_{i} = Q1\left( X \right)Q2\left( Y \right)Q3\left( H \right)$$

In Eq. 12, $w_{i}$ is out coming signal of second layer’s node. Also, *Q* denotes the incoming signals from membership functions (MFs). Additionally, X, Y and H represent location of computing nodes in different directions^19,23^.

In Eq. 13, the signals could be defined in relative form, and as shown in the equation $\overline{{w_{i} }}$ is normalized firing strengths.

13 $$\overline{{w_{i} }} = \frac{{w_{i} }}{{\sum \left( {w_{i} } \right)}}$$

And finally, for obtaining the output from the AI equations, the normalized firing strengths could be considered as a coefficient of output. So, based on the inputs and modified coefficients that could be obtained at the end of AI, as well as the firing strengths, the output can be obtained. The fourth layer is used as the function of a consequence if–then rule that was initiated by Takagi and Sugeno^26^. Therefore, the node function is as follows:

14 $$\overline{{w_{i} }} f_{i} = \overline{{w_{i} }} \left( {p_{i} X + q_{i} Y + r_{i} Z + s_{i} } \right)$$

In which *p*_*i*_, *q*_*i*_, *r*_*i,*_ as well as *s*_*i*_ are the if–then rules' parameters, which could be called consequent parameters^20^.

CFD method has particular features that can limit the studies. It is expensive, complicated, and time-consuming to use. Therefore, artificial intelligence (AI) and particularly ANFIS could be beneficial without using CFD. Moreover, the sensitivity study for the results has not been checked for different inputs for the current model.

## Results and discussion

For training the CFD data set, 70% of all data were used in the learning process, and the rest of the dataset (30%) were kept for testing and validation evaluations. In each stage of analysis, the comparison between the CFD and machine learning data set was considered.

All CFD datasets are randomly selected before training process. This randomize selection enables us to remove connectivity between input parameters. For the evaluation of the training model, 30% of datasets are selected in testing process. In this step, again there is no connectivity between input and output parameters, and the process of testing is fully independent of training datasets. Moreover, different parameters were used in the process, and considerable the time was spent for finding the effective parameter in the study.

**Figure 1:**
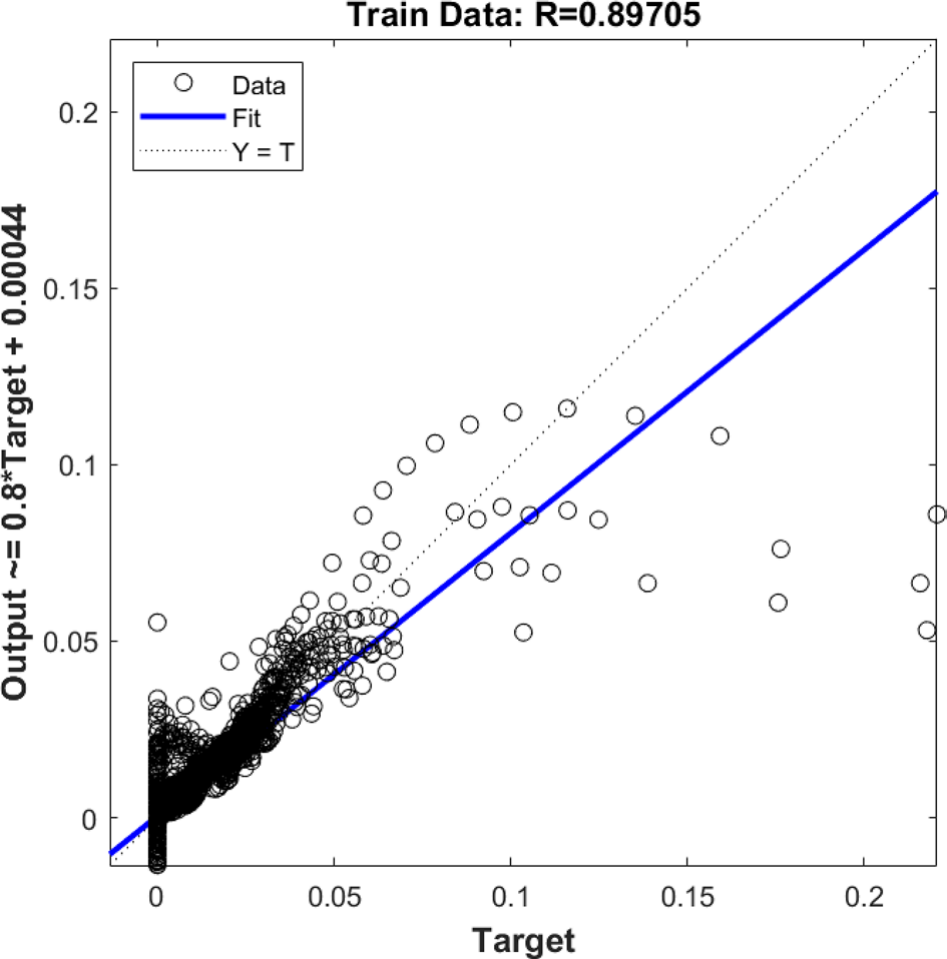
Training assessment for four membership functions in each input parameter.

After running the CFD method, flow characteristics for each computing element were saved in the memory of the computer for the purpose of learning steps. The location of each computing element was considered as the input parameter of the model, and the fraction of gas bubbles in the reactor was set as an output parameter. At the beginning of modeling, four *gaussmf* type membership functions were considered for each input of the learning step. By running simulation, the machine learning results were compared with the CFD finding. The results showed that the value of *R* was not very high, and this value showed a low level of accuracy and, in other words, the low capability of prediction for the model (Fig. 1).

**Figure 2:**
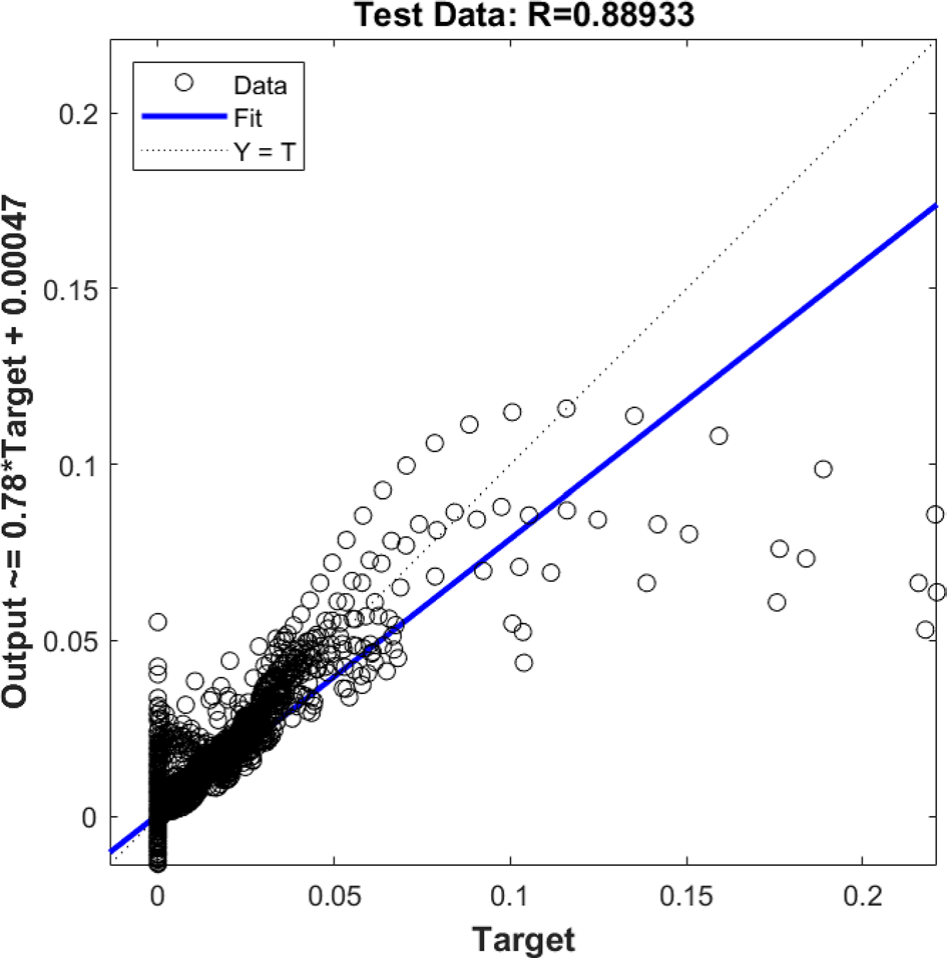
Testing assessment for four membership functions in each input parameter.

Also, the rest of the results were examined, and they were compared with the CFD dataset. Figure 2 demonstrates the testing assessment for four membership functions in each input parameter. In this stage of modeling, the machine learning method did not know about 30% of the dataset, and this model could estimate the rest of the data (remaining 30%) only based on the training step. By comparing results of the CFD and machine learning methods, four membership functions were used in each input, and therefore, the same results were achieved in the training process, and still, the level of accuracy and prediction capability was very low (Fig. 2). After examination of *R* for training and testing, the amount of gas was compared at different computing nodes for CFD and the machine learning method. Four functions in each input parameter are not sufficient modeling tools to translate learning datasets to the FIS structure. Therefore, there is no linear trend between target and model output. For better liner connection between AI model and CFD results, more membership functions are required in each input parameter.

**Figure 3:**
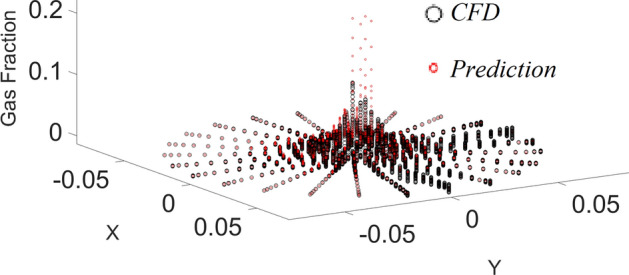
CFD pattern comparison with AI pattern. Red colour shows CFD and black dot shows AI data.

Figure 3 shows that the machine learning algorithm can correctly estimate the amount of gas at some locations of the reactor, particularly near the walls. However, near the sparger region, this model is not very capable in the prediction of gas. The machine learning model with four membership functions is probably not enough complex to find out the complexity of gas sparging in the bubble column reactor.

**Figure 4:**
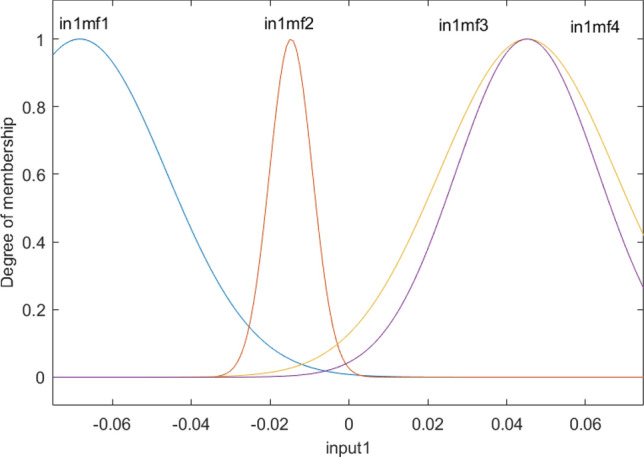
Degree of membership function, and the function performance as a function of the first input for four membership functions for each input parameter.

Figure 4 shows the degree of membership function as a function of x computing nodes. Functions 3 and 4 show almost similar results, and they can cover more range of input 1, while membership 2 can only cover a short range of input 1. This calculation could be analyzed for the second input, and similar behavior could be observed in the second input. This shows that both input one and input two contain almost a similar impact on the fraction of gas in the domain. As these two inputs present, the cross-section of the reactor and gas fraction has a circular shape in each level of reactor similar behavior of MF for inputs 1 and 2 makes sense. As the fraction of gas can be changed in different levels, we analyze the degree of MF versus the level of reactor or z computing nodes.

**Figure 5:**
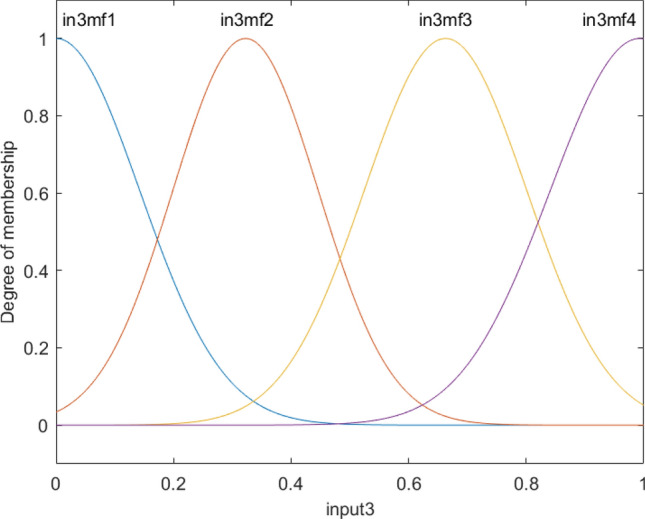
Degree of membership function, and the function performance as a function of the third input for four membership functions for each input parameter.

**Figure 6:**
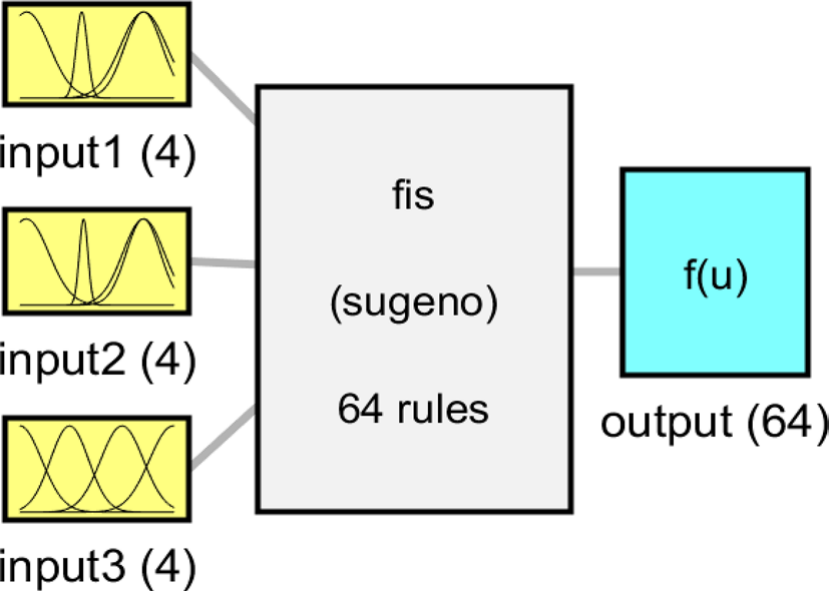
Overall shape of *Fis* structure and membership performance for each input parameter.

Figure 5 shows that all membership functions are uniformly distributed at different levels of the reactor, which is completely different from input 1 and input 2. These results represent the difference in the level of complexity between different inputs, and the first and second inputs can be merged into one input as they have a similar pattern of the degree of membership. The overall shape of functions in each input is shown in Fig. 6.

**Figure 7:**
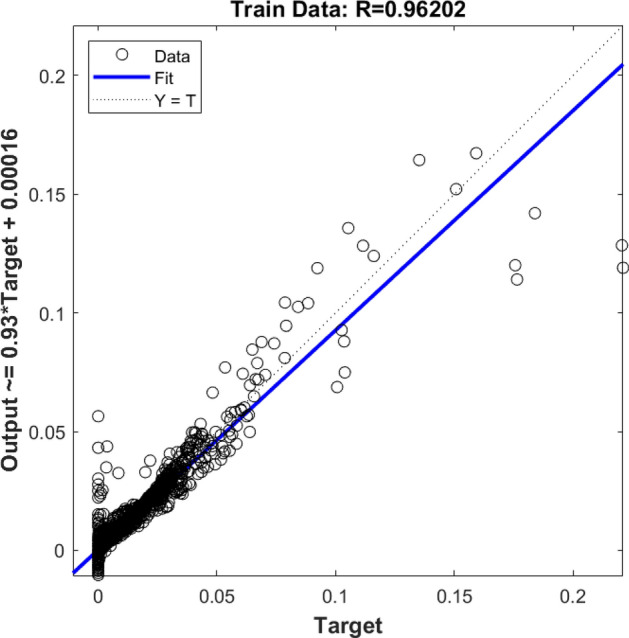
Training assessment for six membership functions in each input parameter.

**Figure 8:**
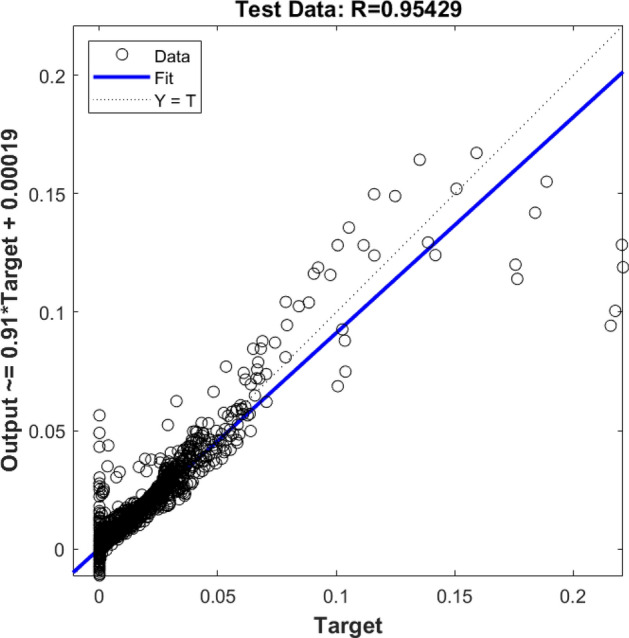
Testing assessment for six membership functions in each input parameter.

**Figure 9:**
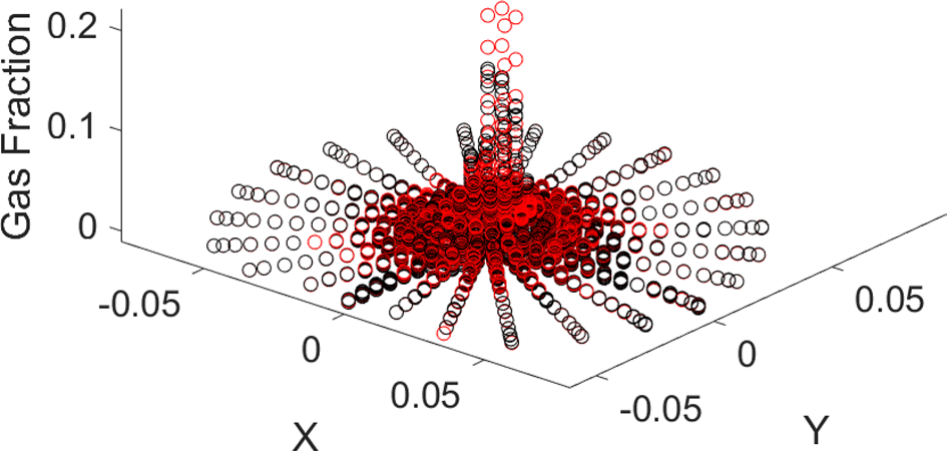
CFD pattern comparison with AI pattern. The red color shows CFD, and the black dot shows AI data.

Additionally, the results showed that the functionality of input 1 and input 2 might be changed by adding more membership functions in each input. For this matter, six membership functions were considered in each input, and again the evaluation of the machine learning model was started. Figures 7, 8, and 9 show the comparisons between the CFD and machine learning methods when six functions were considered in inputs 1, 2, and 3. The results showed that the level of accuracy and the prediction capability significantly increased by increasing membership functions in each input. For a better understanding of the impact of adding the membership function, the degree of membership function was plotted as a function of inputs 1, 2, and 3.

**Figure 10:**
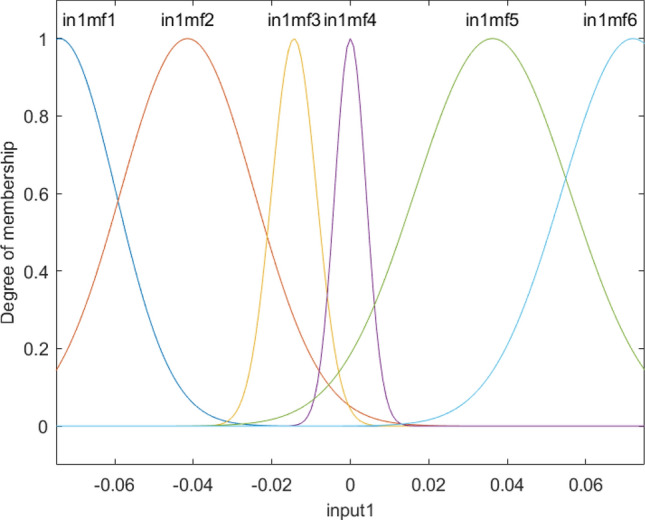
Degree of membership function and the function performance as a function of the first input for six membership functions for each input parameter.

**Figure 11:**
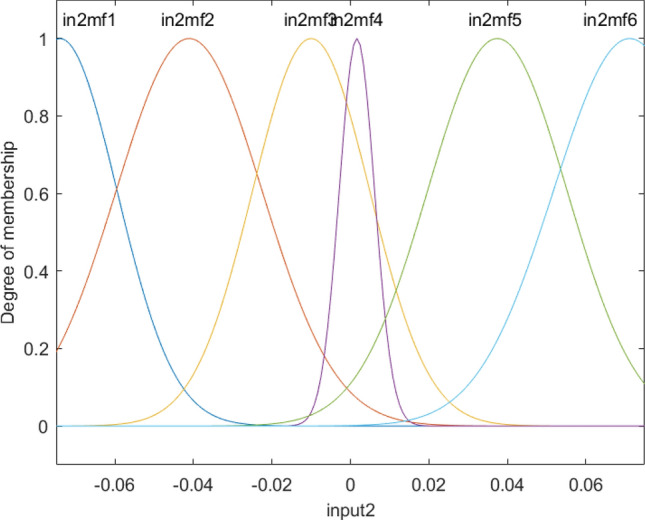
Degree of membership function, and the function performance as a function of the second input for six membership functions for each input parameter.

**Figure 12:**
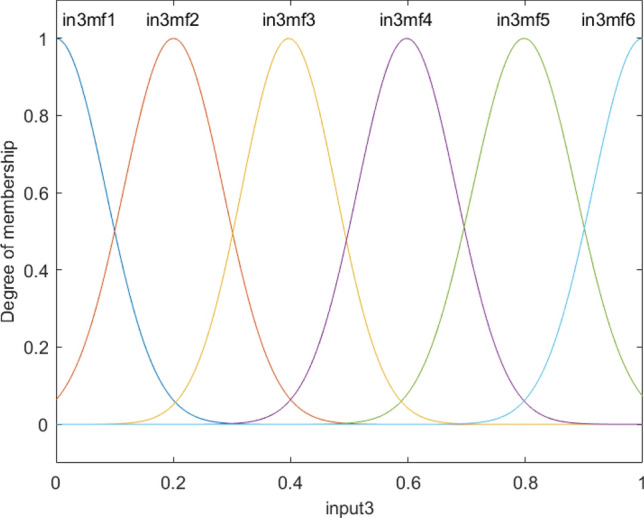
Degree of membership function, and the function performance as a function of the third input for six membership functions for each input parameter.

Figures 10, and 11 showed that for input 1 and 2, membership functions were distributed better than the case of using four functions. However, this function distribution was not similar to z computing nodes. All functions, in this case, had similar behavior across the domain (Fig. 12).

**Figure 13:**
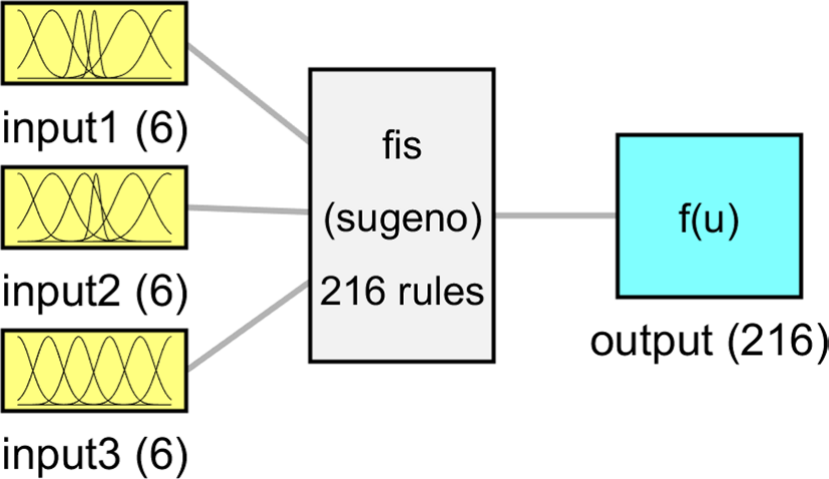
Overall shape of *Fis* structure and membership performance for each input parameter with six membership functions.

The final structure of the machine learning framework is illustrated in Fig. 13. The figure shows that for each input, there were six membership functions. The figure also shows the total number of rules in the model, which is 216 rules. This model structure can be a better representative of the machine learning model to simulate gas fraction in the 3D(three-dimensional) reactor. The connection between input and output parameters and the impact of each input on flow characteristics is beneficial for understanding different types of process engineering and optimizing the operating conditions in two phase reactor^27^. All parameters associated with one process can have a different influence on the final product and efficiency of the equipment. These parameters are tightly coupled together when the operator selects them together.

## Conclusions

The complexity of gas and liquid interaction inside the bubble column reactor can impact the understanding of the process and optimization of these types of reactors in the chemical engineering industry. Numerical methods based on the calculation of Navier–Stokes equations can provide many computed parameters, such as gas fraction, pressure, or turbulence characteristics. However, these parameters require significant statistical and engineering analyses to understand the correlation between them. On the other hand, machine learning methods have emerged better to understand the process behind one engineering process. Therefore, there is a player rule for these algorithms to facilitate process understanding and finding a mathematical correlation between input and output parameters. As such, in this study, the researchers considered the impact of the input parameter on the output parameter of one process called “*bubbly flow*”. In this case, the 3D two-phase bubble column reactor was simulated with the CFD (Eulerian approach), and computed results were participated in the machine learning method to investigate the degree of membership function and complexity of parameters in the reactor. Different membership function patterns for each input of training were considered. All locations of computing nodes (mesh), such as X, Y, and Z elements, had participated in the process of training campaign, and the output for training was gas fraction or gas hold-up. The researchers of the study evaluate each model by training and testing processes, and the influence of membership structure/pattern was investigated for each input parameter. The results showed that there was no significant difference between the evaluation of training and testing processes for all number of membership functions. The results also illustrated the increasing number of membership functions in each input could improve the accuracy and prediction capability of the model. The number of membership functions can also define the complexity of each input and its effect on flow characteristics. Besides, the impact on output parameters could be recognized when the degree of membership function was analyzed as a function of input parameters. This analysis can be useful during the design or optimization of bubbly processes in bubble column reactors. The results showed that X and Y computing nodes have very similar behaviour to impact of gas fraction in the column, while different levels of bubble column reactor contained uniform distribution of membership functions, and this factor was different from inputs 1 and 2. In previous studies, the researchers investigated the effect of inputs on the output. However, the current study sought to investigate which input had the highest effect on the output. The effective parameter can be found based on the impact of MFs on the output, and how the behaviour of each function changes according to the input. Therefore, the degree of MFs was considered as a function of each input parameter.
